# Supplementary material for: Primulina anisocymosa (Gesneriaceae), a new species with a unique inflorescence structure from Guangdong, China
Source: PeerJ. 2019 Jan 4;7:e6157. doi: 10.7717/peerj.6157 (PMC6322489; doi:10.7717/peerj.6157)
Supplement: Appendix S1 — List of the 184 Gesneriaceae samples included in the phylogenetic analysis, with voucher number and deposition, origin information and respective GenBank accession numbers (trnL-F and ITS1/ITS2,), or with citations for previously published sequences). For samples with an RBGE accession number the DNA samples came from cultivated material which was based on the collection numbers given. [file peerj-07-6157-s002.docx]

APPENDIX 1. List of the 184 Gesneriaceae samples included in the phylogenetic analysis, with voucher number and deposition, origin information and respective GenBank accession numbers (*trnL-F* and ITS1/ITS2,), or with citations for previously published sequences). For samples with an RBGE accession number the DNA samples came from cultivated material which was based on the collection numbers given.

***Aeschynanthus lancilimbus*** W.T. Wang. China. unknown locality, Y.Z. Wang S-10868, (PE), FJ501499, HQ632992. ***Aeschynanthus rhododendron*** Ridl. Peninsular Malaysia, Genting Highlands, P. Woods 600 [Cult. RBGE 19680624] (E), HQ632895, FJ501333. ***Aeschynanthus roseoflorus*** Mendum. Indonesia. Seram, G.Argent 87/14 (E), HQ632896, HQ632993. ***Agalmyla bilirana*** Hilliard & B.L.Burtt. Philippines. Leyte Island, RBGE-PNHE 1999-12 (E), HQ632891, HQ632988. ***Agalmyla glabra*** (Merr.) Hilliard & B.L.Burtt. Philippines. Camiguin Island, RBGE-PNHE 1999 -28 (E), HQ632892, HQ632989. ***Agalmyla paucipilosa*** Hilliard & B.L.Burtt. Indonesia. Sulawesi, Mt Rantemario Smith & Galloway 261 (E), HQ632893, HQ632990. ***Agalmyla sojoliana*** Hilliard & B.L.Burtt. Indonesia. Sulawesi, Mt Sojol Smith & Galloway 321 (E), HQ632894, HQ632991. ***Allocheilos guangxiensis*** H.Q.Wen, Y.G.Wei & S.H.Zhong. China. Guangxi, Yongfu county, Y.G.Wei 06-02 (IBK), HQ632897, HQ632994. ***Allostigma guangxiense*** W.T.Wang, M.Möller. China. Guangxi, Longzhou county, MMO 05-755 (E), HQ632880, HQ632977. ***Anna ophiorrhizoides*** (Hemsl.) B.L.Burtt & R.A.Davidson. China. Sichuan, Emei Shan, M.Möller MMO 08-1280 (E), HQ632937, HQ633034. ***Anna submontana Pellegr*** China. Yunnan, Maguan county, M.Möller MMO 01-85 (E, WU), FJ501542, FJ501362. ***Boea hygrometrica*** (Bunge.) R.Br. China. unknown locality, Z.J.Gu 01-6184 (KUN, E), FJ501476, FJ501319. ***Boea philippensis*** C.B.Clarke. Indonesia. Sulawesi, Gunung, Ali S.Scott 02-142 (E), HQ632862, HQ632953. ***Boeica multinervia*** K.Y.Pan. China. Yunnan, Yingjiang, Y.Z.Wang 015 (PE), HQ632861, HQ632951. ***Briggsia mihieri*** (Franch.) Craib. China. Chongqing, Nanchuan county, Y.Z.Wang 11315 (B, PE), FJ501544, FJ501363. ***Briggsiopsis delavayi*** (Franch.) K.Y.Pan. China. Chongqing, Nanchuan county, W.Fang 1 (IBK), HQ632879, HQ632976. ***Cathayanthe biflora*** Chun. China. Hainan, Tongshi county, M.Möller MMO 08-1327 (E), HQ632899, HQ632996. ***Codonoboea albomarginata*** (Hemsl.) Kiew. Peninsular, Malaysia, Perak, Maxwell's Hill, A.Weber 840805-1/12 (WU), AJ492297, HQ632961. ***Codonoboea codonion*** (Kiew.) C.L.Lim. Malaysia. Terengganu, Jerangau, F.R.C.L.Lim FRI 65040 (KEP), JF912538, JF912565. ***Codonoboea corrugata*** (Mendum.) D.J.Middleton. Philippines. Palawan, RBGE-PNHE 1998 s.n. (E), FJ501484, HQ632962. ***Codonoboea elata*** (Ridl.) Rafidah. Malaysia. Perak, Maxwell’s, A.R.Rafidah FRI 64321 (KEP), JF912523, JF912550. ***Codonoboea floribunda*** (M.R.Hend.) C.L.Lim. Malaysia. Terengganu, Sg. Nipah F.R., C.L.Lim FRI 64971(KEP), JF912539, JF912566. ***Codonoboea malayana*** (Hook.f.) Kiew. Malaysia. Pahang, Fraser’s Hill, R.Kiew & D.J.Middleton FRI 57513 (KEP), JF912541, JF912568. ***Codonoboea pumila*** (Ridl.) C.L.Lim. Malaysia. Pahang, Fraser’s Hill, T.L.Yao FRI 55963 (KEP), JF912543, JF912570. ***Codonoboea racemosa*** (Jack.) A.Weber. Indonesia. Sumatra, Aceh, P.S.Smith, SMTSU 110/110 (E), JF912544, JF912571. ***Codonoboea venusta*** (Ridl.) Kiew. Malaysia. Fraser’s Hill, R.Kiew RK 5430 (KEP), JF912545, JF912572. ***Conandron ramondioides*** Siebold & Zucc. Japan. unknown locality, Takeda Herbal Garden, Kyoto [Cult. RBGE 19691267] (E), FJ501515, FJ501340. ***Cyrtandra cumingii*** C.B.Clarke. Japan. Ruykyus, Iriomote Island, G.Kokubugata 11134 (TNS), HQ632905, HQ633002. ***Cyrtandra glabra*** Banks ex C.F.Gaertn. French. Polynesia, Society Is.: Tahiti: Mt. Tearoa Col, Q.C.B.Cronk & D.Percy T91 (E), AY423136, FJ501353. ***Cyrtandra longifolia*** (Wawra.) Hillebr. ex C.B.Clarke. USA. Hawaii, Kauai, M.Kiehn 920825-2/1 [Cult. HBV] (WU), FJ501531, EU919939. ***Cyrtandra pendula*** Blume. Peninsular Malaysia. Negeri Sembilan, Kuala Pilah distr., Jeram Toi, A.Weber & Anthonysamy 860730-1/2 [Cult. HBV] (WU), FJ501530, FJ501354. ***Cyrtandra pulchella*** O.Rich ex A.Gray. Samoa Islands, Lorence 8525 (PTBG), HQ632906, EU919941. ***Damrongia fulva*** (Barnett.) D.J.Middleton & A.Weber. Thailand. Khampaeng Phet, P.Triboun s.n. (BK), JF912536, JF912563. ***Damrongia lacunosa*** (Hook.f.) D.J.Middleton & A.Weber 1 Peninsular Malaysia. Perak, Temengor F.R., Pulau Batu Putih K.Imin et al. FRI 63238 (KEP), JF912530, JF912557. ***Damrongia lacunosa*** (Hook.f.) D.J.Middleton & A.Weber 2 Peninsular Malaysia. Pahang, Lipis distr., Gua Rusa, Weber 870510-1/8 (WU), FJ501458, FJ501308. ***Damrongia purpureolineata*** Kerr ex Craib 1 Thailand. Lamphun, Li P.Triboun s.n. (BK), JF912534, JF912561. ***Damrongia purpureolineata*** Kerr ex Craib 2 Thailand. Lamphun, Li D.J. Middleton et al. 4812 (E), JF912535, JF912562. ***Didymocarpus antirrhinoides*** A.Weber. Peninsular Malaysia.Perak, Bujong Melaka, Ipoh., K.Jong 9009 [Cult. RBGE 19650167] (E), FJ501513, DQ912671. ***Didymocarpus citrinus*** Ridl. Peninsular Malaysia. Perlis, Kedah Peak, P.Davis 69437 [Cult. RBGE 19830510] (E), AJ492293, DQ912669. ***Didymocarpus cordatus*** Wall. ex A.DC. Peninsular Malaysia. Perak, Maxwell’s Hill, A.Weber 860816-2/1 (WU), AJ492294, DQ912673. ***Didymocarpus podocarpus*** C.B.Clarke. Bhutan. Deothang District, H.Noltie, Pradhan, Sherub & Wangdi 193 (E), FJ501514, DQ912688. ***Didymocarpus stenanthos*** C.B.Clarke. China. Yunnan, Binchuan county, M.Möller MMO 01-156 (E, WU), FJ501512, DQ912687. ***Didymocarpus villosus*** D.Don. Nepal. Sundarijal, B.Adhikari SB 9 (E), HQ632904, HQ633001. ***Didymostigma obtusum*** (C.B.Clarke.) W.T.Wang. China. Guangdong, Fengkai county, M.Möller MMO 08-1310 (E), HQ632875, HQ632971. ***Emarhendia bettiana*** (M.R.Hend.) Kiew, A.Weber & B.L.Burtt. Peninsular Malaysia. R.Kiew FRI 55716 (KEP), HQ632864, HQ632955. ***Gyrocheilos chorisepalus*** W.T.Wang var. *synsepalus* W.T.Wang. China. Guangdong, Xinyi county, Y.G.Wei 07-708 (IBK), HQ632900, HQ632997. ***Gyrocheilos lasiocalyx*** W.T.Wang. China. Guangxi, Guiping county, M.Möller MMO 06-881 (E), HQ632901, HQ632998. ***Gyrocheilos retrotrichus*** W.T.Wang. China. Guangxi, Wuming county, M.Möller MMO 07-1136 (E), HQ632902, HQ632999. ***Gyrocheilos retrotrichus*** W.T.Wang var. *oligolobus* W.T.Wang. China. Guangxi, Rongshui county, Sirong town, Y.G.Wei 06-208 (E), HQ632903, HQ633000. ***Hemiboea bicornuta*** (Hayata) OhwiVoucher from Cult. unknown origin, RBGE 19951207 (E), FJ501534, FJ501356. ***Hemiboea cavaleriei*** H.Lév. China. unknown locality, Z.J.Gu G3 (KUN), FJ501533, FJ501355. ***Hemiboea fangii*** Chun ex Z.Yu Li. China. Sichuan, Emei Shan, M.Möller MMO 08-1284 (E), HQ632882, HQ632979. ***Hemiboea follicularis*** C.B.Clarke. China. Guangxi, Huanjiang county, Y.G.Wei G03 (IBK), HQ632885, HQ632982. ***Hemiboea longgangensis*** Z.Yu Li. China. Guangxi, Longzhou county, Y.G.Wei 07-550 (IBK), HQ632889, HQ632986. ***Hemiboea longzhouensis*** W.T.Wang. China. Guangxi, Longan county, M.Möller MMO 07-1127 (E), HQ632888, HQ632985. ***Hemiboea magnibracteata*** Y.G.Wei & H.Q.Wen. China. Guangxi, introduced from Huanjiang county to Guilin city, M.Möller MMO 08-1347 (E), HQ632887, HQ632984. ***Hemiboea omeiensis*** W.T.Wang. China. Sichuan, Emei Shan, M.Möller MMO 08-1271 (E), HQ632886, HQ632983. ***Hemiboea ovalifolia*** (W.T.Wang) A.Weber & Mich.Möller. China. Guangxi, Napo county, Nonghua, B.M.Nong 06-1 (IBK), HQ632883, HQ632980. ***Hemiboea purpureotincta*** (W.T.Wang) A.Weber & Mich.Möller. China. Guangxi, Tian Ling county, M.Möller MMO 06-813 (E), HQ632884, HQ632981. ***Hemiboea rubribracteata*** Z.Yu Li & Yan Liu. China. Guangxi, introduced from Jingxi to Guilin city, M.Möller MMO 07-1093 (E), HQ632890, HQ632987. ***Hemiboea subcapitata*** C.B.Clarke. China. Chongqing, Chengkou county, Y.Z.Wang 11306 (PE), FJ501535, FJ501357. ***Henckelia anachoreta*** (Hance) D.J.Middleton & Mich.Möller. Thailand. Chiang Mai, Doi Suthep, D.J.Middleton et al. 4480 (E), HQ632870, HQ632966. ***Henckelia bifolia*** (D.Don) A.Dietr. Nepal. Chyalding, near Sybrubesi Bhaskar, Adhikari L2B6 (E), JF912522, JF912549. ***Henckelia dielsii*** (Borza) D.J.Middleton & Mich.Möller. China. Yunnan, Jingdong county, M.Möller MMO 08-1211 (E), HQ632871, HQ632967. ***Henckelia floccosa*** (Thwaites) A.Weber & B.L.Burtt. Sri Lanka. C.G.Jang s.n. [G 157] (WU), FJ501486, HQ632964. ***Henckelia grandifolia*** A.Dietr. China. Yunnan, Jingdong county M.Möller MMO 08-1222 (E), JF912527, JF912554. ***Henckelia incana*** (Vahl) Spreng. India. Nilghiri mts, S.Vogel SVG s.n. (E), HQ632869, HQ632965. ***Henckelia longisepala*** (H.W.Li) D.J.Middleton & Mich.Möller. China. Yunnan, Jinping county, Y.M.Shui 73170 (KUN), HQ632890, HQ632963. ***Henckelia pumila*** (D.Don) A.Dietr. 1 Thailand. Chiang Mai, Doi Inthanon D.J. Middleton et al. 4505 (E), JF912529, JF912556. ***Henckelia pumila*** (D.Don) A.Dietr. 2 China. Yunnan, Nujiang Lisu Aut. Pref., Fugong county, Gaoligong Shan Expedition 1996 7938 [Cult. RBGE 19962271] (E), FJ501491, FJ501327. ***Henckelia urticifolia*** (D.Don) A.Dietr. 1 China. Yunnan, J.M.Li 05851 (PE), DQ872821, DQ872835. ***Henckelia urticifolia*** (D.Don) A.Dietr. 2 Bhutan. Tashigang distr. NPSW 110 (E), JF912532, JF912559. ***Henckelia urticifolia*** (D.Don) A.Dietr. 3 Nepal. Sankhuwasabha distr., Arun valley EMAK 109 H (Edinburgh-Makalu Expedition 1991) (E), FJ501492, FJ501328. ***Henckelia walkerae*** (Gardner) D.J.Middleton & Mich.Möller. Sri Lanka; leg. in US 11.03.1996, L.Skog 7736 (US 590934) [Cult. Smithsonian 94-250] (US), FJ501490, FJ501326. ***Hexatheca fulva*** C.B.Clarke. Sarawak. Bau, Fairy Cave, J.Sang & C.Geri S99358 (E), HQ632873, HQ632969. ***Kaisupeea herbacea*** (C.B.Clarke) B.L.Burtt. Thailand. Chachoengsao, Khao Tak Groep,K.Larsen 44272 [Cult. RBGE 19972918] (E), FJ501459, FJ501309. ***Loxocarpus argenteus*** B.L.Burtt. Malaysia. Sarawak, Bako National Park, T.L.Yao FRI 57975 (KEP), JF912537, JF912564. ***Loxocarpus violoides*** (C.B.Clarke) T.L.Yao. Malaysia. Sayap, Kinabalu Park, T.L.Yao FRI 65458 (KEP), JF912546, JF912573. ***Loxostigma glabrifolium*** D.Fang & K.Y.Pan. China. Guangxi, Napo county, Y.G.Wei 709 (IBK), HQ632910, HQ633006. ***Loxostigma griffithii*** (Wight) C.B.Clarke. Nepal. Yamphudin, Kew/Edinburgh, Kanchenjunga Expedition (1989) 940 [Cult. RBGE 19892473A] (E), FJ501508, FJ501338. ***Lysionotus chingii*** Chun ex W.T.Wang. China. unknown locality, Y.Z.Wang S-10669 (PE), FJ501498 , FJ501332. ***Lysionotus pauciflorus*** Maxim. China. Yunnan, Xichou county, Cheng Jia Po, M.Möller MMO 01-101 (E, WU), FJ501497, FJ501331. ***Lysionotus petelotii*** Pellegr. China. Yunnan, road to Xichou, M.Möller MMO 01-100/4 (E), FJ501496, HQ632974. ***Metapetrocosmea peltata*** (Merr. & Chun) W.T.Wang. China. Hainan, Wuzhi Shan, Y.G.Wei 07-702 (IBK), HQ632872, HQ632968. ***Microchirita caliginosa*** (C.B.Clarke) Yin Z. Wang. Peninsular Malaysia ex HB München-Nymphenburg. M.Kiehn & M.Pfosser 2000-1 [Cult. HBV GS-96-02] (WU), FJ501488, FJ501325. ***Microchirita hamosa*** (R.Br.) Yin Z. Wang. China. Guangxi, Longzhou county, M.Möller MMO 05-753 (E), JF912524, JF912551. ***Microchirita involucrata*** (Craib) Yin Z. Wang. Peninsular Malaysia. Kedah, Baling, K. Imin et al. FRI 63180 (KEP), JF912526, JF912553. ***Microchirita lavandulacea*** (Stapf) Yin Z. Wang. China. unknown locality Voucher from Cult. RBGE 20000897 (E), FJ501487, FJ501324. ***Microchirita mollissima*** (Ridl. ) A. Weber & D.J.Middleton. Thailand. Surat Thani, Khlong Phanom D. J. Middleton et al. 4361 (E), JF912528, JF912555. ***Microchirita tubulosa*** (Craib) A. Weber & D. J. Middleton. Thailand. Nakhon Sawan, Wat Thep Satha Phon D. J. Middleton et al. 4809 (E), JF912531, JF912558. ***Microchirita viola*** (Ridl. ) A. Weber & Rafidah. Malaysia. Kedah, P. Langkawi A. R. Rafidah. FRI 64388 (KEP), JF912533, JF912560. ***Orchadocarpa lilacina*** (Ridl. ) Peninsular Malaysia. Pahang, Fraser's Hill R. Kiew, RK 5410 (KEP), HQ632863, HQ632954. ***Oreocharis jiangxiensis*** (W. T. Wang) Mich. Möller & A. Weber. China. Fujian, Jiangle county, M. Möller MMO 09-1451(E), HQ632914, HQ633029. ***Oreocharis acaulis*** (Merr. ) Mich. Möller & A. Weber. China. Guangdong, Zhaoqin county, M. Möller MMO 08-1328 (E), HQ632916, HQ633012. ***Oreocharis argyreia*** Chun ex K. Y. Pan. China. Guangxi, Wuming county, M. Möller MMO 07-1131 (E), HQ632919, HQ633015.

***Oreocharis aurea*** Dunn. China. Yunnan, Jinping county, M. Möller MMO 06-980 (E), HQ632920, HQ633016. ***Oreocharis auricula*** (S. Moore) C. B. Clarke. China. Guizhou, Jiangkou county, M. Möller MMO 03-304 (E), FJ501481, FJ501323. ***Oreocharis begoniifolia*** (H. W. Li) Mich. Möller & A. Weber. China. Yunnan, Jing Dong county, M. Möller MMO 08-1221 (E), HQ632929, HQ633025. ***Oreocharis concava*** (Craib) Mich. Möller & A. Weber. China. Yunnan, Binchuan county, M. Möller MMO 01-153 (E, WU), FJ501505, FJ501336. ***Oreocharis convexa*** (Craib) Mich. Möller & A. Weber. China. Yunnan, Dali county, M. Möller MMO 01-176 (E, WU), FJ501506, FJ501337. ***Oreocharis cotinifolia*** (W. T. Wang) Mich. Möller & A. Weber. China. Guangxi, Dayaoshan, Jinxiu county, Q. M. Chuan 01 (IBK), HQ632914, HQ633010. ***Oreocharis craibii*** Mich. Möller & A. Weber. China. Sichuan, Pan Zhi Hua county, M. Möller MMO 07-1072 (E), HQ632921, HQ633017. ***Oreocharis dasyantha*** Chun var. ferruginosa K. Y. Pan. China. Hainan, Delong Y.G.Wei 07-700 (E), HQ632918, HQ633014. ***Oreocharis esquirolii*** H. Lév. China. Guizhou, An Long county, Longtoushan D. W. Zhang 723 (IBK), HQ632915, HQ633011. ***Oreocharis lancifolia*** (Franch. ) Mich. Möller & A. Weber. China. Sichuan, Mianning county, M. Möller MMO 09-1624 (E), HQ632924, HQ633020. ***Oreocharis longifolia*** (Craib) Mich. Möller & A. Weber. China. Yunnan, Jingdong county, M. Möller MMO 08-1239 (E), HQ632934, HQ633030. ***Oreocharis lungshengensis*** (W. T. Wang) Mich. Möller & A. Weber. China. Guangxi, Longsheng county, M. Möller MMO 06-916 (E), HQ632917, HQ633013.

***Oreocharis magnidens*** Chun ex K. Y. Pan. China. Guangxi, Jinxiu county, M. Möller MMO 06-896 (E), HQ632930, HQ633026. ***Oreocharis mileensis*** (W. T. Wang) Mich. Möller & A. Weber. China. Yunnan, Shilin county, Y.M.Shui 65214 (KUN), HQ632928, HQ633024. ***Oreocharis muscicola*** (Diels) Mich. Möller & A. Weber. unknown origin Kew (1995-2229) (K), FJ501548, FJ501366. ***Oreocharis pankaiyuae*** Mich. Möller & A. Weber. China. unknown locality Voucher from Cult. RBGE 20060865 (E), HQ632925, HQ633021. ***Oreocharis primuliflora*** (Batalin) Mich. Möller & A. Weber. China. Sichuan, Danba county, M. Möller MMO 09-1605 (E), HQ632923, HQ633019. ***Oreocharis primuloides*** (Miq. ) Benth. & Hook. f. ex C. B. Clarke. Japan. unknown locality T.Tsuzuki s. n. [Cult. RBGE 19842178A] (E), FJ501546, FJ501364. ***Oreocharis ronganensis*** (K. Y. Pan) Mich. Möller & A. Weber. China. Guangxi, Rong An county, M. Möller MMO 06-776 (E), HQ632927, HQ633023. ***Oreocharis rosthornii*** (Diels) Mich. Möller & A. Weber. China. Guizhou, Jiangkou Xian Sino-American Bryological Expedition, no. 398 (US 229325) (US), FJ501547, FJ501365. ***Oreocharis sinensis*** (Oliv. ) Mich. Möller & A. Weber. China. Guangdong, Bolou county, M. Möller MMO 08-1329 (E), HQ632912, HQ633008. ***Oreocharis sinohenryi*** (Chun) Mich. Möller & A. Weber. China. Guangxi, Fangcheng county, M. Möller MMO 07-1150 (E), HQ632913, HQ633009. ***Oreocharis stewardii*** (Chun) Mich. Möller & A. Weber. China. Guangxi,Shanjiang county, M. Möller MMO 06-917 (E), HQ632926, HQ633022. ***Oreocharis urceolata*** (K. Y. Pan) Mich. Möller & A. Weber. China. Sichuan, Liangshan Yizu county, M. Möller MMO 09-1633 (E), HQ632922, HQ633018. ***Oreocharis xiangguiensis*** W. T. Wang & K. Y. Pan. 1 China. Guangxi, Lin Gui county, M. Möller MMO 05-741 (E), HQ632932, HQ633028. ***Oreocharis xiangguiensis*** W. T. Wang & K. Y. Pan. 2 China. Guangxi, Longsheng county, M. Möller MMO 06-915 (E), HQ632931, HQ633027. ***Ornithoboea arachnoidea*** (Diels) Craib. Thailand. Chiang Mai, Doi Chiang Dao Voucher from Cult. RBGE 19972903 (E), FJ501461, FJ501312. ***Ornithoboea wildeana*** Craib. China. Yunnan, Xichou county, Y. Z. Wang 00401 (PE) FJ501462, FJ501313. ***Paraboea acutifolia*** (Ridl. ) B. L. Burtt . Peninsular Malaysia. Kedah, Pulau Langkawi, Bukit Terbak A. Weber 86805-2/1 (WU), FJ501464, FJ501314. ***Paraboea birmanica*** (Craib) C. Puglisi. China. Guangxi, Jingxi county, M. Möller MMO 06-862 (E), HQ632866, HQ632958. ***Paraboea capitata*** (Ridl. ) Peninsular Malaysia. Perak, Kinta district A. Weber 870522-5/2 [Cult. HBV] (WU), AJ492298, FJ501315. ***Paraboea crassifolia*** (Hemsl. ) B. L. Burtt. China. Yunnan, Maguan county, M. Möller MMO 01-83 (E, WU), FJ501472, FJ501318. ***Paraboea glandulosa*** (B. L. Burtt) C. Puglisi. Thailand. Kanchanaburi, Thong Pha Phum, Ti Pugae D. J. Middleton & P. Triboun 5202 (BK, E), HQ632867, HQ632959. ***Paraboea umbellata*** (Drake) B. L. Burtt. China. Guangxi, Napo county, M. Möller MMO 01-147 (E, WU), FJ501470, FJ501317. ***Petrocodon ainsliifolius*** W. H. Chen & Y. M. Shui. China. Yunnan, Maguan county, Y. M. Shui et al. 44071 (KUN), HQ632941, HQ633038. ***Petrocodon coccinea*** (C. Y. Wu ex H. W. Li) Yin Z. Wang. China. Guangxi, Napo county, M. Möller MMO 01-141 (E, WU), FJ501516, FJ501365. ***Petrocodon coriaceifolius*** (Y. G. Wei) Y. G. Wei & Mich. Möller. China. Guangxi, Yangshuo county, M. Möller MMO 06-913 (E), HQ632943, HQ633040. ***Petrocodon dealbatus*** Hance. China. Guangdong, Lianxian county, Q.J.Xie J-042 (US 422841) (US), FJ501537, FJ501358. ***Petrocodon ferrugineus*** Y. G. Wei. China. Guangxi, Xincheng county, M. Möller MMO 06-784 (E), HQ632946, HQ633043. ***Petrocodon hancei*** (Hemsl.) A. Weber & Mich. Möller. China. Guangxi, He Zhou city, M. Möller MMO 08-1342 (E), HQ632944, HQ633041.

***Petrocodon hechiensis*** (Y. G. Wei, Yan Liu & F. Wen) Y. G. Wei & Mich. Möller. China. Guangxi, Hechi city, M. Möller MMO 07-1077 (E), HQ632942, HQ633039. ***Petrocodon integrifolius*** (D. Fang & L. Zeng) A. Weber & Mich. Möller. China, Guangxi, Longzhou county, M. Möller MMO 06-865 (E), HQ632940 , HQ633037. ***Petrocodon lui*** (Yan Liu & W. B. Xu) A. Weber & Mich. Möller. China. Guangxi, Jingxi county, Wuping town, Xunma village Y.G.Wei 8012 (IBK) HQ632938, HQ633035. ***Petrocodon scopulorum*** (Chun) Yin Z. Wang. China. Guizhou, Xiuwen county, Maochong village, W.Fang 2010-02 (IBK) HQ632947, HQ633044. ***Petrocodon tiandengensis*** (Yan Liu & B. Pan) A. Weber & Mich. Möller. China. Guangxi, Tiandeng county, M. Möller MMO 07-1164 (E), HQ632945, HQ633042. ***Petrocodon viridescens*** W. H. Chen, Mich. Möller & Y. M. Shui. China. Yunnan, Maguan county, Y. M. Shui et al. 82661 (E), HQ632939, HQ633036. ***Petrocosmea kerrii*** Craib. unknown origin Voucher from Cult. RBGE 19715592 (E), FJ501502, FJ501334. ***Petrocosmea nervosa*** Craib. China. N Yunnan, Smithsonian Institute 78-057 [Cult. RBGE 19933232] (E, US), AJ492299, FJ501335. ***Primulina anisocymosa*** F. Wen, Xin Hong and Z.J. Qiu. China. Guangdong: Gaozhou city F.Wen 20121120 (IBK, ANU), this study. ***Primulina anisocymosa*** F. Wen, Xin Hong and Z.J. Qiu. China. Guangdong: Yangchun, *Fang Wen* *201090406-1* (IBK); this study. ***Primulina bipinnatifida*** (W. T. Wang) Yin Z. Wang. China. Guangxi, (PE), DQ872806, DQ872842. ***Primulina*** ***bobaiensis*** Li, Pan and Zhang. China. Yulin City: Bobai County. *F. Wen 2014081001* (IBK); this study. ***Primulina cordifolia*** (D. Fang & W.T. Wang) Yin Z. Wang. China. Guangxi J.M.Li 05561 (PE), DQ872803, DQ872845. ***Primulina dryas*** (Dunn) Mich. Möller & A. Weber. China. Hong Kong T.C.Godfrey 369 [Cult. RBGE 19791050] (E), FJ501524, FJ501348. ***Primulina gemella*** (D. Wood) Yin Z. Wang Vietnam, Hong Quang Special Region, Cat Hai L.Averyanov 1987 [Cult. RBGE 19941913] (E), FJ501523, FJ501345. ***Primulina glandulosa*** (D. Fang et al. ) Yin Z. Wang var. yangshuoensis (F. Wen, Q. X. Zhang & Yue Wang) Mich. Möller & A. Weber. China. Guangxi, Yang Shuo county, M. Möller MMO 06-912 (E), HQ632948, HQ633045. ***Primulina glandulosa*** (D. Fang et al. ) Yin Z. Wang. China. Guangxi, J. M. Li 054291 (PE), DQ872804, DQ872841. ***Primulina heterotricha*** (Merr. ) Yin Z. Wang. China. Guangxi, Y. Z. Wang 067311 (PE), DQ872816, DQ872826. ***Primulina linearifolia*** (W. T. Wang) Yin Z. Wang . China. Guangxi, J. M. Li 11121 (PE), DQ872810, DQ872834. ***Primulina longgangensis*** (W. T. Wang) Yin Z. Wang. Vietnam. unknown locality, A. Takhtajan & N. Aruzytov 1975 [Cult. RBGE 19941915] (E), AJ492290, FJ501347. ***Primulina luochengensis*** (Yan Liu & W. B. Xu) Mich. Möller & A. Weber. China. Guangxi, Luocheng county, Xiaochangan town, M.Möller MMO 07-1163 (E), HQ632949, HQ633046. ***Primulina minutimaculata*** (D. Fang et W. T. Wang) Yin Z. Wang. China. Guangxi, J. M. Li 067134 (PE), DQ872815, DQ872828. ***Primulina mollifolia*** (D. Fang et W. T. Wang) Yin Z. Wang. China. Guangxi, J. M. Li 054281 (PE), DQ872802, DQ872847. ***Primulina ophiopogoides*** (D. Fang et W. T. Wang) Yin Z. Wang. China. Guangxi, Y. Z. Wang 067134 (PE), DQ872814, DQ872829. ***Primulina pinnata*** (W. T. Wang) Yin Z. Wang. China. Guangxi, Rongshui Xian Expedition Beijing 896526 (US 294374) (US), FJ501526, FJ501349. ***Primulina pinnatifida*** (Hand.-Mazz. ) Yin Z. Wang. China. Guangdong, Lianxian county, Q.J.Xie J-037 (US 422838) (US), FJ501527, FJ501350. ***Primulina pteropoda*** (W. T. Wang) Yin Z. Wang. China. Guangxi, Y. Z. Wang 067312 (PE), DQ872817, DQ872827. ***Primulina repanda*** (W. T. Wang) Yin Z. Wang var. guilinensis (W. T. Wang) Mich. Möller & A. Weberex. China. Guangxi, Smithsonian Institute 94-083 [Cult. RBGE 19951206] (E), AJ492292, FJ501351. ***Primulina spadiciformis*** (W. T. Wang) Mich. Möller & A. Weberex. China. unknown locality, Smithsonian Institute 94-087 [Cult. RBGE 19951205] (E), AJ492291, FJ501346. ***Primulina spinulosa*** (D. Fang et W. T. Wang) Yin Z. Wang. China. Guangxi, Y. Z. Wang 067133 (PE), DQ872813, DQ872830. ***Primulina tabacum*** Hance. China. Guangdong, Lian River Q.J. Xie & C.X. Ye s.n. [Cult. RBGE 19951540] (E), AJ492300, FJ501352. ***Primulina weii*** Mich. Möller & A. Weber. China. Guangxi J. M. Li, Ljm-04-42 (PE), DQ872811, DQ872832. ***Primulina wentsaii*** (D. Fang et L. Zeng) Yin Z. Wang. China. Guangxi, J. M. Li 11630 (PE), DQ872812, DQ872831. ***Pseudochirita guangxiensis*** (S. Z. Huang) W. T. Wang. China. Guangxi, Mashan county, M. Möller MMO 06-798 (E), HQ632908, HQ633003. ***Pseudochirita guangxiensis*** (S. Z. Huang) W. T. Wang var. glauca Y. G. Wei & Yan Liu. China. Guangxi, Jingxi county, M. Möller MMO 05-751 (E), HQ632909, HQ633004. ***Raphiocarpus sinicus*** Chun. China. Guangxi, Shangsi county, M. Möller MMO 07-1141 (E), HQ632877, HQ632973. ***Ridleyandra petiolata*** (Ridl. ) A. Weber. Peninsular Malaysia. G. Inas M.A.Mohd. Hairul FRI 60092 (KEP), HQ632935, HQ633032. ***Ridleyandra porphyrantha*** (A. Weber & Kiew) A. Weber. Malaysia. Pahang, side ridge of Gunung Bunga Buah A. Weber 870420-2/4 (WU), FJ501520, HQ633031. ***Ridleyandra quercifolia*** (Ridl. ) A. Weber. Peninsular Malaysia. Perak, Maxwell Hill, T.L.Yao FRI 65405 (KEP), HQ632936, HQ633033. ***Senyumia minutiflora*** (Ridl. ) Kiew, A. Weber & B. L. Burtt. Peninsular Malaysia. Pahang, Gunung Senyum, A.R.Rafidah, R. Kiew & M. A. Mohd. Hairul FRI 55722 (KEP), HQ632865, HQ632957. ***Spelaeanthus chinii*** Kiew, A. Weber & B. L. Burtt. Peninsular Malaysia. Pahang, Jerantut distr, Taman Negara A. Weber 860709-2/2 (WU), FJ501457, FJ501307. ***Streptocarpus andohahelensis*** Humbert. Madagascar. Tuléar, Ranomafana, M. Möller MM 9717 (E), FJ501449, AF316903. ***Streptocarpus beampingaratrensis*** Humbert. Madagascar. Tuléar, Ranomafana, M. Möller MM 9715 (E), FJ501448, AF316905. ***Streptocarpus dunnii*** Hook. f. Swaziland, Mbabane I. La Croix s. n. [Cult. RBGE 19941745] (E), FJ501456, AF316951. ***Streptocarpus hilsenbergii*** R. Br. Madagascar. Mandrake valley, B. L. Burtt s. n. [Cult. RBGE 19631505] (E), FJ501450, AF316907. ***Streptocarpus holstii*** Engl. Tanzania. unknown locality, Cornell University (Bail. Hort) [Cult. RBGE 19592272] (E), AJ492304, AF316917. ***Streptocarpus ibityensis*** Humbert. Madagascar. Antananarivo, E. Fischer 250/93 [Cult. RBGE 19932867] (E), FJ501455, AF316926. ***Streptocarpus papangae*** Humbert. Madagascar. Tuléar, Ranomafana, M. Möller MM 9718 (E), FJ501444, AF316929. ***Streptocarpus rexii*** (Hook.) Lindl. South Africa. NE Cape, Grahamstown, K. Jong s. n. [Cult. RBGE 19870333] (E), AJ492305, AF316979. ***Tetraphyllum roseum*** Stapf. Thailand. Krabi Province, H. K. Kurzweil 798 (WU), FJ501434, HQ632950.
